# Plasma metabolite biomarkers for multiple system atrophy and progressive supranuclear palsy

**DOI:** 10.1371/journal.pone.0223113

**Published:** 2019-09-27

**Authors:** Akio Mori, Kei-Ichi Ishikawa, Shinji Saiki, Taku Hatano, Yutaka Oji, Ayami Okuzumi, Motoki Fujimaki, Takahiro Koinuma, Shin-Ichi Ueno, Yoko Imamichi, Nobutaka Hattori

**Affiliations:** Department of Neurology, Juntendo University School of Medicine, Tokyo, Japan; Karolinska Institutet, SWEDEN

## Abstract

Radiological biomarkers have been reported for multiple system atrophy and progressive supranuclear palsy, but serum/plasma biomarkers for each disorder have not been established. In this context, we performed a pilot study to identify disease-specific plasma biomarkers for multiple system atrophy and progressive supranuclear palsy. Plasma samples collected from 20 progressive supranuclear palsy patients, 16 multiple system atrophy patients and 20 controls were investigated by comprehensive metabolome analysis using capillary electrophoresis mass spectrometry and liquid chromatography mass spectrometry. Medication data were obtained from patients with multiple system atrophy and progressive supranuclear palsy, and correlations with associated metabolites were examined. Receiver operating characteristics curve analyses were used to investigate diagnostic values for each disorder. The levels of 15 and eight metabolites were significantly changed in multiple system atrophy and progressive supranuclear palsy, respectively. Multiple system atrophy was mainly characterized by elevation of long-chain fatty acids and neurosteroids, whereas progressive supranuclear palsy was characterized by changes in the level of oxidative stress-associated metabolites. Receiver operating characteristic curve analyses revealed that patients with multiple system atrophy or progressive supranuclear palsy were effectively differentiated from controls by 15 or 7 metabolites, respectively. Disease-specific metabolic changes of multiple system atrophy and progressive supranuclear palsy were identified. These biomarker sets should be replicated in a larger sample.

## Introduction

Multiple system atrophy (MSA) and progressive supranuclear palsy (PSP) are devastating parkinsonian disorders, typically characterized by sporadic adult-onset motor symptoms including gait difficulty, akinesia and increased muscle tone, with limited response to levodopa [1]. Particularly in the early stages, it is often difficult to make accurate diagnoses that differentiate various parkinsonian disorders from idiopathic Parkinson’s disease (PD) [2]. The forms of α-synucleinopathy include PD with neuronal cytoplasmic inclusions (Lewy bodies) and MSA with glial cytoplasmic inclusions (GCIs), whereas PSP is a tauopathy with neuronal cytoplasmic tau accumulation [3, 4]. For the development of disease-modifying therapies to treat each disorder, it is important to achieve accurate diagnosis of each disease using simple, accessible, and cost-effective methods [5]. In this context, several previous studies have suggested the use of biomarkers based on cerebrospinal fluid (CSF) or serum/plasma analysis. Low levels of CSF/plasma coenzyme Q10 are reported to be significantly decreased in MSA [6, 7]. In MSA and PSP, neurofilament light chain levels in both the CSF and the serum are reported to be significantly increased compared with controls [8, 9]. Although plasma levels of α-synuclein, DJ-1, and total tau in MSA are reported to be altered compared with controls [10], at present, no clinically established serum/plasma biomarkers for differential diagnosis between MSA and PSP have been identified [3,4].

Comprehensive metabolome analysis has recently been developed as a useful tool for biomarker identification based on understanding the pathophysiological changes associated with neurodegenerative disease [3,10,11]. In the current study, we performed plasma metabolome analysis using a dual method combining liquid chromatography time-of-flight mass spectrometry (LC-TOFMS) with capillary electrophoresis time-of-flight mass spectrometry (CE-TOFMS) to investigate potential biomarkers for parkinsonian disorders. Fifteen metabolites in MSA and eight metabolites in PSP were significantly changed, and each set of metabolites was revealed as a novel disease-specific biomarker, suggesting that they may each be implicated in a disease-specific pathogenesis.

## Materials and methods

### Study population

This study protocol complied with the Declaration of Helsinki and was approved by the ethics committee of Juntendo University (2012157). Written informed consent was obtained from all participants. Participants were recruited from the Department of Neurology at Juntendo University Hospital in Tokyo, Japan. We enrolled 20 healthy controls, 16 MSA patients, and 20 PSP patients without any history of cancer, aspiration pneumonia, type 2 diabetes mellitus or collagen vascular diseases (Table 1). Diagnosis of MSA patients was made using the “Second consensus statement on the diagnosis of MSA” [12], and diagnosis of PSP patients was made using the National Institute for Neurological Disorders and Stroke/Society criteria for PSP [13]. The age at sampling of patients with PSP was significantly greater than that of MSA or controls. There were no significant differences in sex ratio or disease durations between groups, and all patients with MSA and PSP were able to perform gait with assistance.

**Table 1:** Characteristics of the cohort.

	Controls	MSA	PSP	p^a^
**N**	20	16	20	-
**Age (SEM) [years]**	66.2 (2.05)	64.8 (1.76)	72.6 (1.45)^c^	0.0067
**Duration (SEM) [years]**	-	3.56 (0.580)	4.95 (0.510)	0.0594
**Sex (F:M)**	9:11	8:8	7:13	0.646^b^

Abbreviations: MSA = multiple system atrophy; PSP = progressive supranuclear palsy; SEM = standard error of the mean.

^a^p-value obtained by analysis of variance

^b^p-value obtained by chi-square test.

^c^p = 0.0291 compared with control, 0.0039 compared with MSA by Steel-Dwass test.

### Sample collection

The sample collection process was described in our previous report [13]. Briefly, following overnight fasting, a plasma sample was obtained using 7 ml EDTA-2Na blood tubes (PN, SRL). After resting for 30–60 minutes at 4°C, the spots were centrifuged for 10 minutes at 2,660 × g. After the plasma separation, the collected samples were placed in liquid nitrogen and kept until analysis. The metabolome analysis was performed in February 2017.

### Metabolome analysis

The details of this method were described in our previous report [13]. Metabolite extraction and metabolome analysis were performed by Human Metabolome Technologies (HMT) based on methods described previously [14]. Briefly, for CE-TOFMS analysis, 50 μl plasma samples were added to 450 μl methanol containing internal standards (H3304-1002, HMT) on ice, and then mixed with 500 μl chloroform and 200 μl Milli-Q water. The solution was centrifuged at 2,300 × g for 5 min at 4°C and the upper aqueous layer was centrifugally filtered through Millipore 5 kDa cutoff filter (UltrafreeMC-PLHCC, HMT) at 9,100 ×g for 120 min at 4°C to remove macromolecules. The filtrate was then centrifugally concentrated and reconstituted in 25 μl Milli-Q water prior to CE-TOFMS analysis. The analysis was performed using an Agilent CE system equipped with an Agilent 6210 TOFMS, an Agilent 1100 series binary HPLC pump, a G1603A CE-MS adapter kit and a G1607A CE-ESI-MS sprayer kit (Agilent Technologies). These systems were controlled by Agilent G2201AA ChemStation software and connected by a fused silica capillary (50 μm i.d. × 80 cm) filled with commercial electrophoresis buffer (H3301-1001 and H3302-1021 for cation and anion analyses, respectively; HMT). Exact mass data were acquired over a 50–1000 m/z range.

For LC-TOFMS analysis, 500 μl plasma samples were mixed with 1,500 μl acetonitrile with 1% formic acid containing internal standard solution (H3304-1002, HMT) on ice. The solution was centrifuged at 2,300 ×*g* for 5 min at 4°C and the supernatant was applied to a Hybrid SPE phospholipid cartridge (55261-U, Sigma-Aldrich). The filtrate was dried by nitrogen gas and reconstituted in 200 μl of 50% isopropanol. LC-TOFMS analysis was conducted by an Agilent 1200 series RRLC system SL and an Agilent 6230 TOFMS (Agilent Technologies) equipped with ODS column (2 × 50 mm, 2 μm). These systems were controlled by Agilent G2201AA ChemStation software (Agilent Technologies).

Data obtained from both CE-TOFMS and LC-TOFMS were processed by MasterHands (Keio University) for extracting peak information including m/z, peak area, migration time (MT) for CE-TOFMS, and retention time (RT) for LC-TOFMS. Signal peaks corresponding to isotopomers, adduct ions, and other product ions of known metabolites were excluded, and remaining peaks were annotated according to the HMT metabolite database. The annotated peak areas were then normalized based on internal standard levels and sample volumes for relative quantification.

### Statistical analysis

Statistical analysis was carried out using JMP13 (SAS Institute Inc.). To exclude the influence of age and gender, all metabolites were analyzed by multiple regression analysis, followed by Wilcoxon’s test or analysis of covariance to compare MSA or PSP and controls. To visualize the observed metabolomic profile as a heat map representation, we performed hierarchical clustering analyses for each disorder with JMP13. After imputation of missing values with half of the minimum observed value for each metabolite, we used the Steel-Dwass test for comparing each metabolite among the three groups (MSA, PSP, and controls). Receiver operating characteristics (ROC) curve analysis was carried out using JMP12 or 13. The optimal cut-off value and area under the curve (AUC) were calculated.

## Results

### The metabolome datasets

We analyzed the metabolomic profiles of plasma obtained from 20 controls, 16 MSA, and 20 PSP patients using CE-TOFMS and LC-TOFMS. Based on m/z values, migration times and retention times, 123 metabolites were identified in all participants (Supplementary Data S1 File). In addition, 193 metabolites detected in more than 50% of participants were analyzed in detail. In terms of medication-associated metabolites, we excluded 3-methoxytyrosine detected in patients with MSA or PSP treated with levodopa, because its level clearly reflected the medication dose S1 Table.

### Metabolites significantly changed in MSA

The list of significantly changed metabolites is summarized in Table 2. We performed Wilcoxon’s test to identify significantly altered metabolites followed by reconfirmation of the significance under normalized conditions of age at sampling using analysis of covariance. Fifteen metabolites exhibited significant differences in patients with MSA compared with controls using both statistical methods. In MSA, the levels of 11 metabolites were significantly increased, while the levels of four were significantly decreased. Four of these metabolites were fatty acids (FA): FA(14:0), FA(14:1)-1, FA(14:1)-2, and FA(18:0), none of which were identified in PSP. Likewise, neurosteroids like pregnenolone sulfate (Preg-S) and dehydroisoandrosterone 3-sulfate (DHEAS) were significantly increased in MSA (Table 2). Although decreased levels of lysophosphatidylcholine (LysoPC) (16:0) were detected, the results revealed a significant increase in levels of 7-dehydrocholesterol-1.

**Table 2:** Metabolites specific for MSA.

Compound	Canonical pathway	Mean	SEM	Ratio*	p-value**
**FA(14:0)**	Fatty acid metabolism	8.20E-05	6.63E-06	1.41	0.0103
**FA(14:1)-1**	Fatty acid metabolism	6.91E-06	6.18E-06	1.40	0.0184
**FA(14:1)-2**	Fatty acid metabolism	5.59E-06	7.49E-07	1.57	0.0400
**FA(18:0)**	Fatty acid metabolism	9.76E-04	7.00E-05	1.30	0.0314
**LysoPC(16:0)**	Lysolipid	7.38E-06	1.00E-06	0.67	0.00278
**Pregnenolone sulfate**	Neurosteroid	5.10E-05	6.41E-06	2.13	0.001
**DHEAS**	Neurosteroid	1.77E-03	1.70E-04	1.75	0.0027
**Betaine**	Choline metabolism	1.88E-02	1.07E-03	0.78	0.0014
**Ergosterol-1**	Cholesterol metabolism	7.17E-06	5.67E-07	1.40	0.0038
**7-Dehydrocholesterol-1**	Vitamin D3 metabolism	1.90E-05	8.50E-07	1.12	0.0059
**4-Androsten-3,17-dione-2**	Endogenous steroid	1.10E-05	1.82E-06	1.64	0.0261
**Hecogenin**	Intake?	1.46E-04	8.21E-06	0.77	0.0049
**Guanidinosuccinic acid**	Argininosuccinic acid metabolism	1.67E-04	2.51E-05	2.01	0.0014
**Methionine**	Amino acid	4.29E-03	2.30E-04	0.77	0.0017
**Urea**	Urea cycle	6.43E-01	3.71E-02	1.26	0.0078

Abbreviations: FA: fatty acid; LysoPC(16:0): 1-Palmitoyl-glycero-3-phosphocholine; MSA: multiple system atrophy; DHEAS: dehydroisoandrosterone 3-sulfate; SEM: standard error of the mean. Statistical methods: All metabolites were analyzed by multivariate logistic regression to exclude the influence of age and gender.

* The metabolite level ratio of MSA to controls.

**p-value obtained by Wilcoxon’s test, compared with controls.

**Fig 1:**
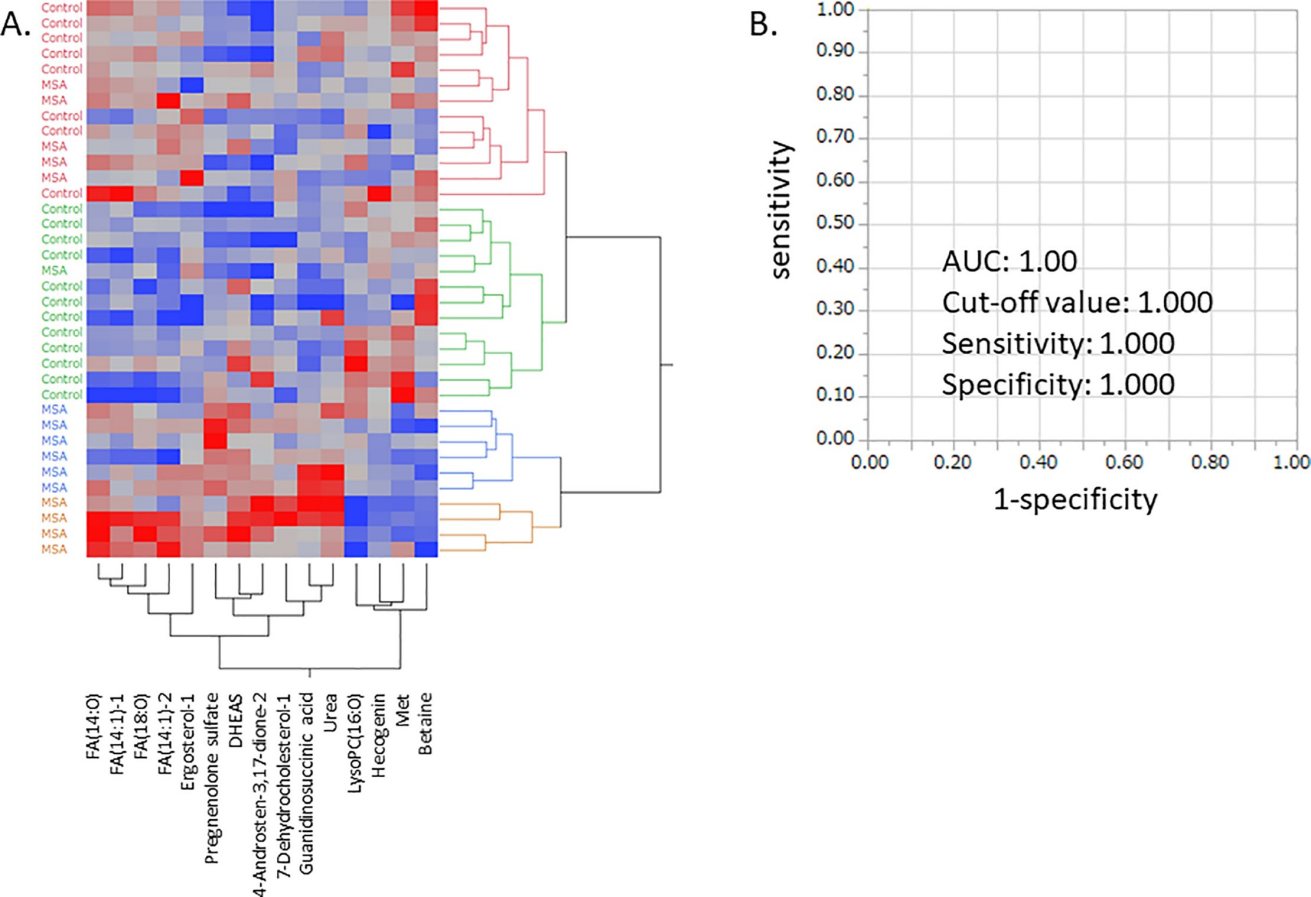
Metabolites showing significant changes in MSA patients compared with controls. Hierarchical clustering analysis was performed. Red indicates higher than average metabolite concentrations, while green indicates below average metabolite concentrations. Abbreviations: MSA: multiple system atrophy; FA: fatty acid; DHEAS: dehydroisoandrosterone 3-sulfate; LysoPC(16:0): lysophosphatidylcholine (16:0); Met: methionine.

Next, we performed hierarchical clustering analysis (HCA) to visualize metabolomic data of controls and MSA. As shown in Fig 1, more than 60% of MSA patients formed a single cluster. To examine whether these metabolites could be useful as diagnostic biomarkers for MSA, we performed ROC curve analysis. The individual AUCs of all 15 metabolites showed good predictive accuracy (AUC > 0.7) (Table 3) and combination of 2 or 3 MSA-specific metabolites with higher significance, Preg-S, guanidinosuccinic acid, and methionine, efficiently discriminated MSA from controls (Fig 1B).

**Table 3:** Diagnostic values of each metabolite specific to MSA.

	AUC	Cut-off value
**FA(14:0)**	0.753	0.463
**FA(14:1)-1**	0.733	0.475
**FA(14:1)-2**	0.703	0.463
**FA(18:0)**	0.713	0.463
**LysoPC(16:0)**	0.717	0.450
**Pregnenolone sulfate**	0.825	0.613
**DHEAS**	0.795	0.575
**Betaine**	0.814	0.475
**Ergosterol-1**	0.786	0.575
**7-Dehydrocholesterol-1**	0.769	0.413
**4-Androsten-3,17-dione-2**	0.719	0.438
**Hecogenin**	0.777	0.475
**Guanidinosuccinic acid**	0.816	0.663
**Methionine**	0.809	0.525
**Urea**	0.753	0.463
**Pregnenolone sulfate +** **Guanidinosuccinic acid**	0.847	0.638
**Pregnenolone sulfate +** **Guanidinosuccinic acid +** **Methionine**	0.931	0.788

Abbreviations: MSA: multiple system atrophy; AUC: area under the curve; FA: fatty acid; LysoPC(16:0): lysophosphatidylcholine (16:0); DHEAS: dehydroisoandrosterone 3-sulfate

### Metabolites significantly changed in PSP

Eight metabolites were significantly different in patients with PSP compared with controls when normalized for age (Table 4). Four metabolites exhibited increased levels, while four exhibited decreased levels. Although significant changes of LysoPC(16:0) and 7-dehydrocholesterol-1 were also detected in MSA, LysoPC(16:0) levels were decreased significantly more in PSP. Five metabolites have been reported to be related to oxidative stress. Symmetric dimethylarginine (SDMA), 5-oxoproline, and cysteine glutathione disulfide were increased, while uric acid and diosgenin-1 were decreased.

**Table 4:** Metabolites specific for PSP.

Compound	Canonical pathway	Mean	SEM	Ratio*	p-value**
**SDMA**	Inhibitor of NO synthase	2.59E-04	1.16E-05	1.18	0.0081
**LysoPC(16:0)**	Lysolipid	6.15E-06	6.79E-07	0.56	0.0009
**5-Oxoproline**	L-glutamic acid metabolism	8.77E-04	3.00E-05	1.18	0.0052
**7-Dehydrocholesterol-1**	Vitamin D3 metabolism	1.90E-05	4.11E-07	1.12	0.0020
**AC(13:1)**	Acyl-CoA metabolism	2.83E-06	3.91E-07	0.67	0.0489
**Cysteine glutathione disulfide**	Glutathione metabolism	2.52E-04	2.69E-05	1.56	0.0109
**Diosgenin-1**	Steroid sapogenin	3.18E-06	4.66E-07	0.63	0.0042
**Uric acid**	Purine nucleotides metabolism	1.72E-02	1.24E-03	0.77	0.0158

Abbreviations: PSP: progressive supranuclear palsy; SDMA: symmetric dimethylarginine fatty acid; AC: acylcarnitine; SEM: standard error of the mean.

Statistical methods: All metabolites were analyzed by multivariate logistic regression to exclude the influence of age and gender.

* The metabolite level ratio of PSP to controls.

**p-value obtained by Wilcoxon’s test, comparing PSP with controls.

**Fig 2:**
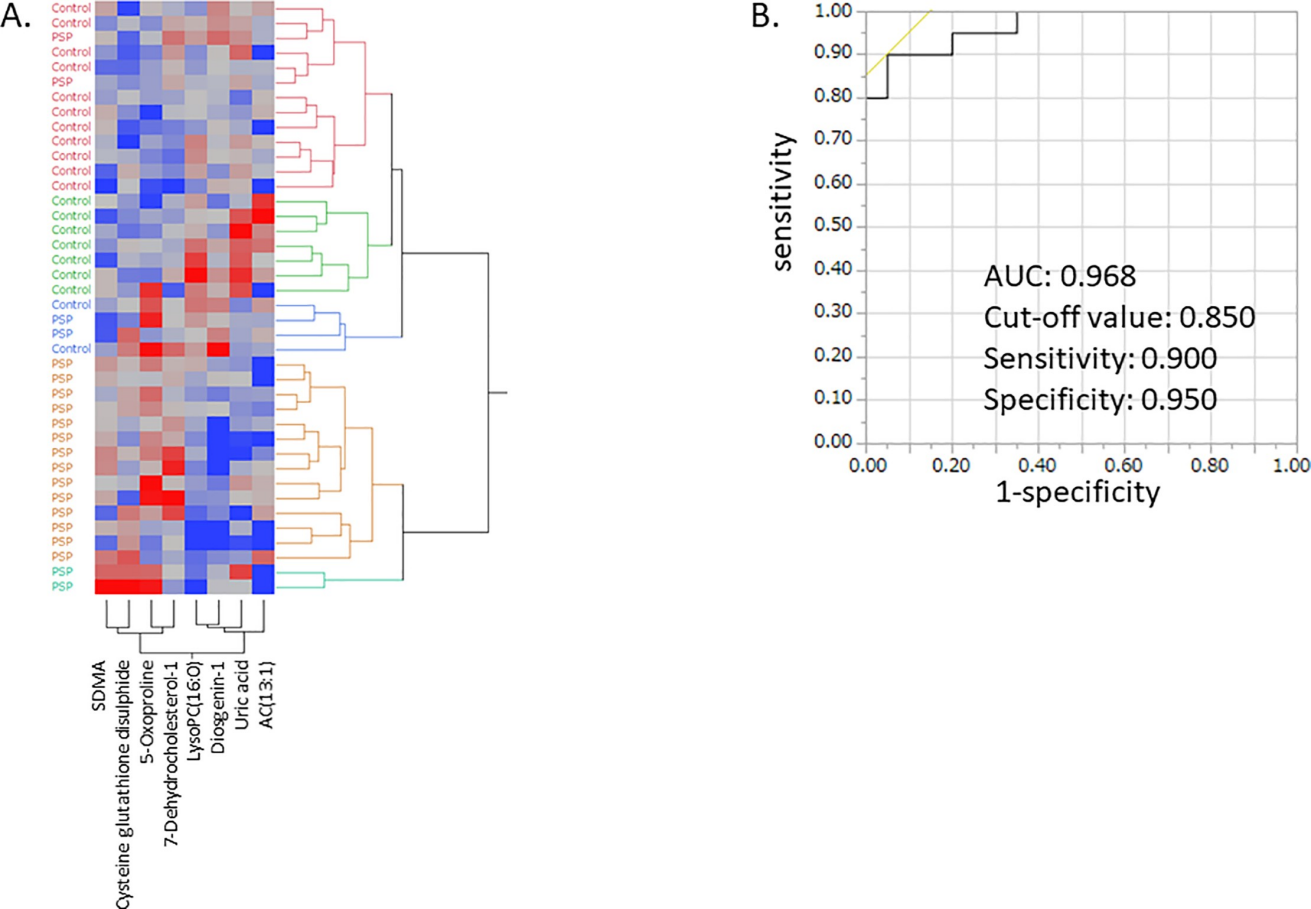
Metabolites showing significant changes in PSP patients compared with controls. Hierarchical clustering analysis was performed. Red indicates higher than average metabolite concentrations, while green indicates below average metabolite concentrations. Abbreviations: PSP: progressive supranuclear palsy; SDMA: symmetric dimethylarginine fatty acid; LysoPC(16:0): lysophosphatidylcholine (16:0); AC: acylcarnitine.

Next, we performed HCA to visualize metabolomic data of controls and PSP. As shown in Fig 2, more than 85% of patients with PSP formed a single cluster based on the levels of the eight significantly changed metabolites, suggesting promising clinical applications of this set of metabolites. ROC analysis for differentiating PSP from controls confirmed that the predictive accuracy was moderate (AUC > 0.7) for seven of eight metabolites, excluding acylcarnitine (AC) (13:1). By using combination of LysoPC(16:0) and 7-Dehydrocholesterol-1 with or without Diosgenin-1, which were top 3 metabolites with higher AUC in this analysis, PSP was distinguished with higher accuracy (Table 5).

**Table 5:** Diagnostic values of each metabolite specific for PSP.

	AUC	Cut-off value
**SDMA**	0.745	0.400
**LysoPC(16:0)**	0.808	0.550
**5-Oxoproline**	0.759	0.500
**7-Dehydrocholesterol-1**	0.783	0.450
**AC(13:1)**	0.681	0.350
**Cysteine glutathione disulfide**	0.736	0.450
**Diosgenin-1**	0.765	0.450
**Uric acid**	0.724	0.450
**P LysoPC(16:0) +** **7-Dehydrocholesterol-1**	0.808	0.4947
**LysoPC(16:0) +** **7-Dehydrocholesterol-1 +** **Diosgenin-1**	0.918	0.747

Abbreviations: PSP: progressive supranuclear palsy; AUC: area under the curve; SDMA: symmetric dimethylarginine fatty acid; LysoPC(16:0): lysophosphatidylcholine (16:0); AC: acylcarnitine

### Differential diagnosis between MSA and PSP

**Fig 3:**
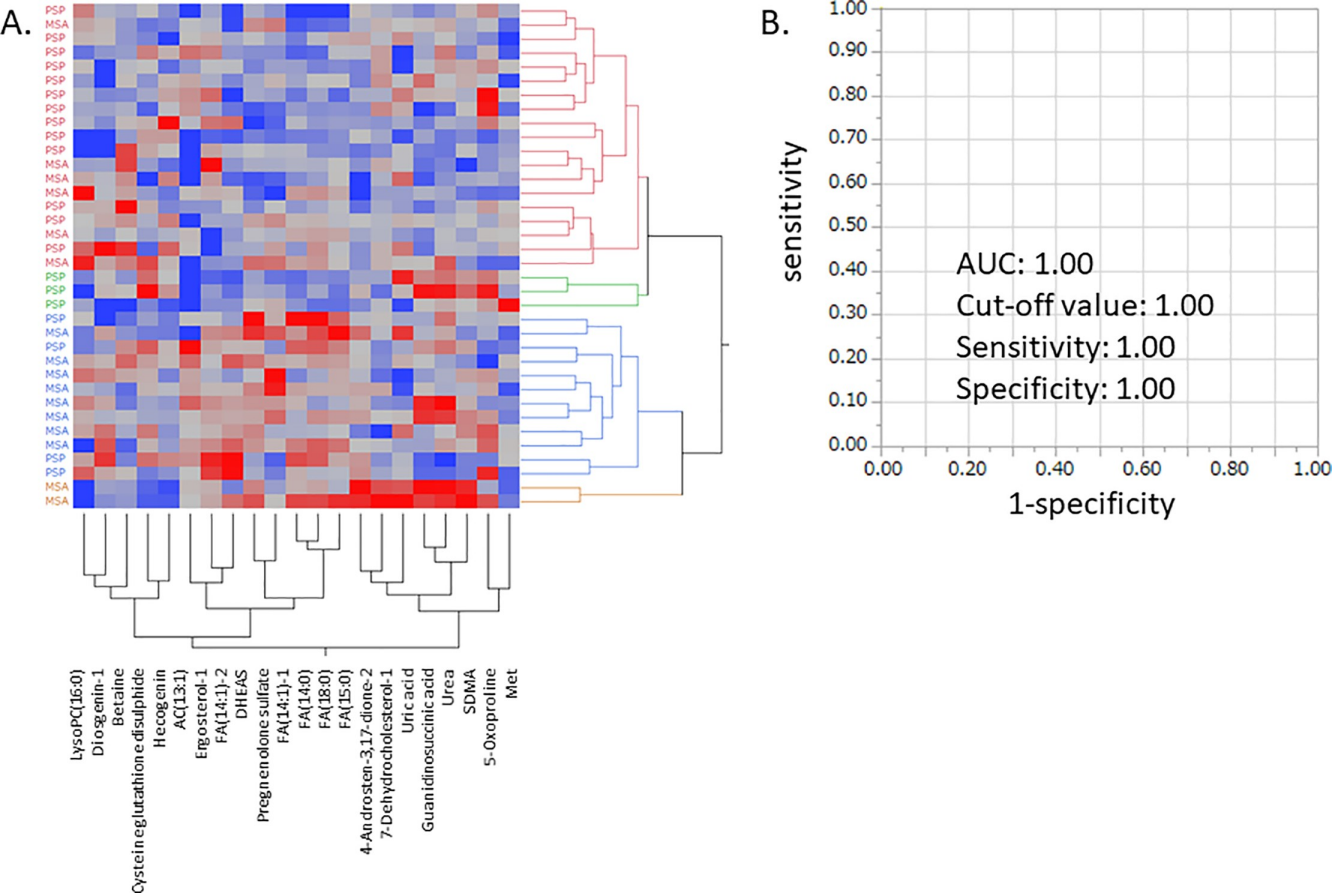
MSA and PSP patients effectively differentiated the disorders using the indicated metabolites. Hierarchical clustering analysis was performed. Red indicates higher than average metabolite concentrations, while green indicates below average metabolite concentrations. Abbreviations: MSA: multiple system atrophy; PSP: progressive supranuclear palsy; AUC: area under the curve; FA: fatty acid; LysoPC(16:0): lysophosphatidylcholine (16:0); DHEAS: dehydroisoandrosterone 3-sulfate; SDMA: symmetric dimethylarginine fatty acid; AC: acylcarnitine; Met: methionine.

We selected 21 metabolites because two metabolites, LysoPC(16:0) and 7-dehydrocholesterol-1, overlapped in both diseases, and investigated the accuracy of differential diagnosis between the two parkinsonian disorders. As shown in Fig 3, most of the upper cluster consisted of PSP patients, while the lower part primarily consisted of MSA patients. ROC curve analysis showed that FA(18:0), preg-S, and DHEAS were effective for differentiating patients with MSA from patients with PSP, and the AUC value for these 3 metabolites revealed that patients with MSA were differentiated from patients with PSP with relatively moderate accuracy (Table 6).

**Table 6:** Diagnostic values of each metabolite specific to MSA differentiating from PSP.

	AUC	Cut-off value
**FA(14:0)**	0.691	0.425
**FA(14:1)-1**	0.697	0.463
**FA(14:1)-2**	0.622	0.413
**FA(18:0)**	0.769	0.563
**LysoPC(16:0)**	0.595	0.313
**Pregnenolone sulfate**	0.734	0.463
**DHEAS**	0.788	0.588
**Betaine**	0.509	0.100
**Ergosterol-1**	0.595	0.375
**7-Dehydrocholesterol-1**	0.556	0.263
**4-Androsten-3,17-dione-2**	0.549	0.188
**Hecogenin**	0.684	0.463
**Guanidinosuccinic acid**	0.597	0.225
**Methionine**	0.634	0.375
**Urea**	0.623	0.263
**SDMA**	0.500	0.213
**5-Oxoproline**	0.591	0.213
**AC(13:1)**	0.575	0.263
**Cysteine glutathione disulfide**	0.622	0.275
**Diosgenin-1**	0.690	0.438
**Uric acid**	0.578	0.263
**DHEAS + FA(18:0)**	0.827	0.550
**DHEAS + FA(18:0) +** **Pregnenolone sulfate**	0.816	0.525

Abbreviations: MSA: multiple system atrophy; PSP: progressive supranuclear palsy; AUC: area under the curve; FA: fatty acid; LysoPC(16:0): lysophosphatidylcholine (16:0); DHEAS: dehydroisoandrosterone 3-sulfate; SDMA: symmetric dimethylarginine fatty acid; AC: acylcarnitine

## Discussion

In the current pilot study, we identified several metabolites as potential biomarkers to distinguish patients with parkinsonian disorders from controls using comprehensive metabolome analysis with a dual separation method. FA and neurosteroids were increased in MSA, and LysoPC(16:0), and oxidative stress markers were changed in PSP. ROC curve analysis using these identified metabolites revealed relatively high AUC values for MSA and PSP. Likewise, MSA patients were distinguished from PSP patients using levels of three metabolites.

We observed significantly increased levels of FAs (FA(14:0), FA(14:1)-1, FA(14:1)-2, and FA(18:0)) in MSA. FA is mainly catalyzed to acyl-coenzyme A (acyl-CoA) by long-chain acyl-CoA synthetase in the mitochondrial outer membrane in the skeletal muscles, but acyl-CoA is unable to penetrate mitochondrial membranes. Acyl-CoA is transformed to acylcarnitine and shuttled into the mitochondrial matrix for degradation via the β-oxidation system by carnitine palmitoyltransferase 1 (CPT1) and carnitine-acylcarnitine translocase (CACT) [15]. Therefore, high levels of FA suggest the disruption of β-oxidation or FA transport across the mitochondrial membrane. Previous studies reported that plasma FA levels were increased in PD [16, 17]. Likewise, we previously reported decreased long-chain acylcarnitine and increased FA in PD, suggesting β-oxidation suppression [13]. In this study, levels of long-chain acylcarnitines were at the lower limit of detection in more than 50% of participants. However, a similar suppression of β-oxidation might occur in MSA.

Two steroid hormones, Preg-S and DHEAS, were found to be increased in MSA in the current study. Preg-S is synthesized from pregnenolone, which is a precursor to all steroid hormones, and DHEAS is produced from DHEA, which occurs upstream of sex hormones in steroidogenesis. These two steroids are classified as neurosteroids, and could modulate synaptic activity [18]. DHEAS has also been reported to have neuroprotective effects against oxidants [19]. Moreover, a previous study using a rodent model reported that pregnenolone sulfate levels are correlated with cognitive performance [20].

Levels of LysoPC(16:0) were decreased in MSA and PSP. Because LysoPC is synthesized from phosphatidylcholine (PC) by the enzyme phospholipase A2, the decrement of LysoPC indicated impairment of these enzymes in PC re-acylation or de-acylation [21]. Because significantly higher levels of 6 LysoPC have been detected in diabetic males [22], it is currently unclear how phospholipid levels in the brain and peripheral blood are related [23]. In patients with Alzheimer’s disease (AD), decreased plasma levels of LyoPC have been reported [24], suggesting that two tauopathies, PSP and AD, might share a disturbance in phosphatidylcholine metabolism, leading to suppression of LysoPC synthesis.

Oxidative stress is one of the major causes of neurodegenerative diseases, including MSA and PSP [25]. Mitochondrial dysfunction and excessive reactive oxygen species (ROS) production in mesenchymal stem cells from patients with PSP have been previously reported [26]. The current results revealed changes in five oxidative stress markers in patients with PSP. Uric acid is considered an antioxidant, and previous studies have indicated that low serum uric acid levels are correlated with the risk of disease and progression in PSP [27]. Cysteine glutathione disulfide is produced by oxidative stress, while 5-oxoproline functions as an oxidant associated with glutathione synthesis, and SDMA, an inducer of oxidative stress in endothelial cells, is upregulated, while the antioxidative effects of diosgenin-1 are downregulated in PSP [28, 29]. Taken together, these results suggest that patients with PSP may be exposed to excessive oxidative stress.

To investigate whether the biomarkers identified in the current study were able to distinguish MSA and PSP from PD, we compared the present data with previous metabolomics data of serum or plasma samples from PD S2 Table [30–37]. Increased FA levels and decreased levels of uric acid have been reported in PD. Although the comparison between the present results and previous findings limited by differences in the measurement methods and conditions used, overall the results suggest several potential biomarkers for distinguishing PD from parkinsonian disorders.

The current study involved several limitations that should be considered. First, the study was performed in a single cohort with a relatively small sample at a single university hospital. In addition, although there were no significant differences in serum levels of creatinine among the three groups (controls vs MSA, p = 0.472; controls vs PSP, p = 0.625 by Wilcoxon’s test), because plasma levels of SDMA are strongly correlated with renal function [38], we cannot completely exclude the possibility that renal function affected SDMA levels in the current study. Because all patients with MSA and PSP in this study exhibited relatively mild conditions, we were unable to evaluate the influence of severity on the levels of metabolites. MSA and PSP have several clinical phenotypes. However, it is difficult to draw conclusions about the relationship between a phenotype and changes of metabolites because the sample size was too small.

In recent years, there have been many attempts to diagnose neurological diseases from blood or other biological samples even in the prodromal stage by metabolomic, proteomic, and transcriptomic analysis, and several candidate biomarkers have been reported. As mentioned in introduction, serum/CSF neurofilament light chain and some other proteins were reported as biomarker of MSA and/or PSP. In the future, we would like to conduct large-scale multi-cohort studies to identify combination biomarkers including proteins and metabolites.

## Conclusions

We identified 15 and 7 metabolites as potential diagnostic plasma biomarkers for MSA and PSP, respectively. Importantly, three of these metabolites were found to be useful for differential diagnosis between the two disorders, and all of the metabolites appeared to beneficial for differentiating the two disorders from PD. Further investigation in larger samples, including PD patients, would be valuable to confirm the clinical utility of these biomarkers.

## Supporting information

S1 Table Effects of levodopa on 3-methoxytyrosine levels in MSA and PSP. Abbreviations: MSA: multiple system atrophy, PSP: progressive supranuclear palsy; SEM: standard error of the mean. *p-value obtained by Wilcoxon’s test, comparing each group with controls.
(DOCX)

S2 Table Comparison of metabolite levels in this study with those of previous reports. Abbreviations: N.A.: Not applicable.
Statistical methods: * The metabolite level ratio of MSA or PSP to controls. **p-value obtained by Wilcoxon’s test, comparing between MSA or PSP and controls.***Data of FA(14:0), (14:1)-1, and (14:1)-2 in this study.
**** Data of FA(18:0) in this study. Highlights indicate statistically significant differences in this study.
(DOCX)

S1 File Supplementary data file. **Showing** sample list, raw data of detected metabolites, clinical parameters, and results of comparative analysis by Steel-Dwass test.
(XLSX)
