# Supplementary material for: Plasma metabolite biomarkers for multiple system atrophy and progressive supranuclear palsy
Source: PLoS One. 2019 Sep 27;14(9):e0223113. doi: 10.1371/journal.pone.0223113 (PMC6764690; doi:10.1371/journal.pone.0223113)
Supplement: S1 Table — Abbreviations: MSA: multiple system atrophy, PSP: progressive supranuclear palsy; SEM: standard error of the mean. *p-value obtained by Wilcoxon’s test, comparing each group with controls. (DOCX) [file pone.0223113.s001.docx]

**Supplementary Table 1. Effects of levodopa on 3-methoxytyrosine levels in MSA and PSP.**

|  |  | **MSA** | | **PSP** | |
| --- | --- | --- | --- | --- | --- |
| **3-methoxytyrosine** | **Mean** | 3.37E-03 | | 5.03E-03 | |
|  | **SEM** | 3.42E-03 | | 8.00E-04 | |
|  | **Ratio to controls** | 218.45 | | 326.00 | |
|  | **p-value** | <0.0001 | | <0.0001 | |
|  |  | **With levodopa** | **Without levodopa** | **With levodopa** | **Without levodopa** |
|  | **Mean** | 4.25E-03 | 1.50E-05 | 5.59E-03 | 1.50E-05 |
|  | **SEM** | 3.33E-03 | 0 | 7.80E-04 | 2.20E-06 |
|  | **Ratio to controls** | 268.87 | 0.9723 | 362 | 0.9723 |
|  | **p-value** | <0.0001 | 0.7963 | <0.0001 | 0.7561 |

Abbreviations: MSA: multiple system atrophy, PSP: progressive supranuclear palsy; SEM: standard error of the mean

*p-value obtained by Wilcoxon’s test, comparing each group with controls.
