# Supplementary material for: Plasma metabolite biomarkers for multiple system atrophy and progressive supranuclear palsy
Source: PLoS One. 2019 Sep 27;14(9):e0223113. doi: 10.1371/journal.pone.0223113 (PMC6764690; doi:10.1371/journal.pone.0223113)
Supplement: S2 Table — Abbreviations: N.A.: Not applicable. Statistical methods: * The metabolite level ratio of MSA or PSP to controls. **p-value obtained by Wilcoxon’s test, comparing between MSA or PSP and controls.***Data of FA(14:0), (14:1)-1, and (14:1)-2 in this study. **** Data of FA(18:0) in this study. Highlights indicate statistically significant differences in this study. (DOCX) [file pone.0223113.s002.docx]

**Supplementary Table 2. Comparison of metabolite levels in this study with those of previous reports.**

|  | **MSA** | | **PSP** | | Reference |
| --- | --- | --- | --- | --- | --- |
|  | **Ratio*** | ***p-value***** | **Ratio*** | ***p-value***** |  |
| **3-Methoxytyrosine** | 2.18E+02 | <0.001 | 3.26E+02 | <0.001 | [13] |
| **Urea** | 1.26 | 0.0078 | 1.11 | 0.189 | [13] |
| **Homovanillic acid** | N.A. |  | N.A. |  | [13] |
| **Guanidinosuccinic acid** | 2.01 | 0.0014 | 1.57 | 0.058 | [13] |
| **Cortisone** | 1.03 | 0.9111 | 1.0 | 0.569 | [13] |
| **Oleoylethanolamine** | N.A. |  | N.A. |  | [13] |
| **Palmitoylethanolamine** | 1.18 | 0.312 | 1.07 | 0.446 | [13] |
| **Citric acid** | 1.04 | 0.712 | 1.05 | 0.446 | [13] |
| **Deoxycholic acid** | 0.83 | 0.577 | 1.25 | 0.499 | [13] |
| **Long-chain acylcarnitines** | 1.02 | >0.05^†^ | 0.67-1.13 | >0.05^†^ | [13] |
| **Medium-long chain fatty acids** | 1.40-1.57 | <0.05^‡^ | 1.11-1.26 | >0.05^‡^ | [13] |
| **Aspartylphenylalanine** | N.A. |  | N.A. |  | [30] |
| **Benzoate** | N.A. |  | N.A. |  | [30] |
| **Serine** | 0.93 | 0.078 | 0.93 | 0.174 | [30] |
| **Inosine** | N.A. |  | 1.05 | 0.342 | [30] |
| **3-hydroxykynuerine,** | N.A. |  | N.A. |  | [11] |
| **Kynurenic acid** | N.A. |  | N.A. |  | [11] |
| **Anthranilic acid** | N.A. |  | N.A. |  | [11] |
| **1-methylhistamine** | N.A. |  | N.A. |  | [31,17] |
| **Alanine** | 0.91 | 0.110 | 0.97 | 0.533 | [32] |
| **Leucine** | 0.97 | 0.774 | 1.17 | 0.357 | [32] |
| **Isoleucine** | 0.99 | 0.873 | 1.23 | 0.227 | [32] |
| **Tryptophan** | N.A. |  | N.A. |  | [33] |
| **Bilirubin** | N.A. |  | N.A. |  | [33] |
| **Ergothioneine** | N.A. |  | N.A. |  | [33] |
| **Biliverdin** | N.A. |  | N.A. |  | [33] |
| **Methionine** | 0.77 | 0.002 | 1.06 | 0.218 | [33] |
| **Threonine** | 0.94 | 0.313 | 1.07 | 0.588 | [34] |
| **Pyroglutamate** | N.A. |  | N.A. |  | [34] |
| **Ketoleucine** | N.A. |  | N.A. |  | [34] |
| **FA(16:0), FA(18:0)** | 1.30^¶^ | 0.031^¶^ | 0.96^¶^ | 0.59^¶^ | [34] |
| **Pyruvate** | 0.87 | 0.238 | 1.01 | 0.705 | [35] |
| **Sorbitol** | N.A. |  | N.A. |  | [35] |
| **Myoinositol** | N.A. |  | N.A. |  | [35] |
| **Ethymalonate** | N.A. |  | N.A. |  | [35] |
| **Propylene glycol** | N.A. |  | N.A. |  | [35] |
| **Suberate** | N.A. |  | N.A. |  | [35] |
| **Methylmalonate** | N.A. |  | N.A. |  | [35] |
| **Galactitol** | N.A. |  | N.A. |  | [35] |
| **Citrate** | 1.04 | 0.712 | 1.05 | 0.446 | [35] |
| **Malate** | 1.02 | 1.00 | 0.99 | 0.925 | [35] |
| **Succinate** | 0.96 | 0.853 | 0.82 | 0.324 | [35] |
| **Glycerol** | 1.03 | 0.678 | 0.94 | 0.394 | [35] |
| **Isocitrate** | 0.98 | 0.535 | 0.96 | 0.645 | [35] |
| **Ethanolamine** | 0.86 | 0.361 | 0.99 | 0.837 | [35] |
| **Ascorbate** | N.A. |  | N.A. |  | [35] |
| **Threonate** | 1.08 | 0.463 | 0.96 | 0.839 | [35] |
| **Gluconate** | 1.21 | 0.886 | 1.17 | 0.188 | [35] |
| **Trimethylamine** | N.A. |  | N.A. |  | [35] |
| **Glutarate** | N.A. |  | N.A. |  | [35] |
| **Methylamine** | N.A. |  | N.A. |  | [35] |
| **Hypoxanthine** | 0.92 | 0.381 | 1.13 | 0.297 | [36] |
| **8-hydroxy-2-deoxyguanosine** | N.A. |  | N.A. |  | [37] |
| **Uric acid** | 0.86 | 0.151 | 0.77 | 0.016 | [37] |
| **Glutathione** | N.A. |  | N.A. |  | [37] |

Abbreviations: N.A.: Not applicable.

Statistical methods: * The metabolite level ratio of MSA or PSP to controls. **p-value obtained by Wilcoxon’s test, comparing between MSA or PSP and controls. ^†^Data of AC(13:1) and (14:1) in this study. ^‡^Data of FA(14:0), (14:1)-1, and (14:1)-2 in this study.

^¶^ Data of FA(18:0) in this study. Highlights indicate statistically significant differences in this study.
